# Silver- and Zinc-Decorated Polyurethane Ionomers with Tunable Hard/Soft Phase Segregation

**DOI:** 10.3390/ijms22116134

**Published:** 2021-06-07

**Authors:** Irene Rapone, Vincenzo Taresco, Valerio Di Lisio, Antonella Piozzi, Iolanda Francolini

**Affiliations:** 1 Department of Chemistry, Sapienza University of Rome, 00185 Rome, Italy; irene.rapone@tiscali.it (I.R.); valerio.dilisio@uniroma1.it (V.D.L.); antonella.piozzi@uniroma1.it (A.P.); 2 School of Chemistry, University of Nottingham, Nottingham NG7 2QL, UK; vincenzo.taresco@nottingham.ac.uk

**Keywords:** polyurethanes, ionomers, metallo-organic hybrid systems, silver, zinc, antimicrobial polymers

## Abstract

Segmented polyurethane ionomers find prominent applications in the biomedical field since they can combine the good mechanical and biostability properties of polyurethanes (PUs) with the strong hydrophilicity features of ionomers. In this work, PU ionomers were prepared from a carboxylated diol, poly(tetrahydrofuran) (soft phase) and a small library of diisocyanates (hard phase), either aliphatic or aromatic. The synthesized PUs were characterized to investigate the effect of ionic groups and the nature of diisocyanate upon the structure–property relationship. Results showed how the polymer *hard*/*soft* phase segregation was affected by both the concentration of ionic groups and the type of diisocyanate. Specifically, PUs obtained with aliphatic diisocyanates possessed a *hard*/*soft* phase segregation stronger than PUs with aromatic diisocyanates, as well as greater bulk and surface hydrophilicity. In contrast, a higher content of ionic groups per polymer repeat unit promoted phase mixing. The neutralization of polymer ionic groups with silver or zinc further increased the *hard*/*soft* phase segregation and provided polymers with antimicrobial properties. In particular, the Zinc/PU hybrid systems possessed activity only against the Gram-positive *Staphylococcus epidermidis* while Silver/PU systems were active also against the Gram-negative *Pseudomonas aeruginosa*. The herein-obtained polyurethanes could find promising applications as antimicrobial coatings for different kinds of surfaces including medical devices, fabric for wound dressings and other textiles.

## 1. Introduction

Segmented polyurethanes (PUs) are considered among the most biocompatible and hemocompatible materials. They are widely used for medical device manufacturing, especially cardiovascular implants, central venous catheters, vascular grafts, heart valves and hemodialysis membranes [1]. This class of polymers possesses a peculiar segregated structure consisting of *hard* and *soft* domains, which not only greatly contributes to the biocompatibility of these polymers but also makes them extremely versatile from a mechanical point of view [2]. Indeed, the mechanical properties of PUs can be considerably varied so that the end-product can be designed according to specific user needs. The key structural parameter in PUs is, therefore, the degree of phase segregation, which depends on several factors, including chemical incompatibility between *hard* and *soft* segments, length of the two segments and *hard*/*soft* phase ratio [3]. Generally, *soft* segments are hydrophilic macrodiols, such as polyester or polyether diols, characterized by a low glass transition temperature, while *hard* segments are aromatic or aliphatic diisocyanates plus low molecular weight diol [1]. The *soft* phase has been shown to be the main responsible for polymer haemo and biocompatibility, in relation to its hydrophilicity features [4] and size of *soft* domains [5]. Strongly hydrophilic *soft* phases have been also shown to reduce bacterial adhesion on PU surfaces [6], with benefits in terms of control of microbial biofilm formation and infection development. 

As far as the *hard* segments are concerned, although they are responsible for the good mechanical resistance and water stability of PUs, they were found to be thrombogenic in platelet retention experiments [7]. The choice of the *hard* phase is, therefore, crucial for PU application in the biomedical field. The introduction of ionic functionalities in the *hard* segment may be considered as a way to modify PU behavior in a biological environment since ionic groups can increase polymer affinity with water [8,9]. In this regard, ionic domains have been shown to reduce the PU thrombogenic potential by affecting platelet and fibrinogen deposition on the polymer surface [10]. Similarly, Mao et al. [11] reported a good blood compatibility of polyurethane ionomer nanoparticles while Filip et al. [12] proposed sodium deoxycholate-based poly(ester-ether)urethane ionomers as antimicrobial biomaterials.

Upon introduction of ionic groups, also the physical properties of PUs can be strongly affected. Indeed, the difference in polarity between the *soft* and *hard* phase upon ionization can act as a driving force for polymer phase segregation, thus improving solid-state properties such as toughness and elastomeric behavior [13,14]. In general, the extent of phase segregation in ionomer PUs depends on the type and number of acidic/basic functions present in the polymer backbone as well as on the nature of the neutralizing cations/anions [15].

Ionic groups can also modulate PU’s ability to conjugate proteins, drugs or biologically active substances. For instance, they can act as ligands for metal ions to obtain inorganic−organic hybrid coordination polymers with defined structures, which are gaining a growing attention in different application fields including biomedicine [16,17,18,19].

Overall, ionomer PUs hold the potential to combine the well-known mechanical and biostability properties of PUs with the strong hydrophilicity of ionomers, further expanding the field of application of PUs. On the basis of this potentiality, ionomer PUs requires a thorough understanding of the structure–property relationship, as well as a continuous updating of knowledge.

Specifically, despite a variety of studies on polyurethane ionomers, there are relatively few publications related to the synthesis of ionomer PUs containing more than one ionic functionality in the *hard* phase [20,21]. Indeed, usually, the ionic monomer, which most of the time is also the chain extender, is added in molar ratio with respect to the diisocyanate-polyol-diisocyanate prepolymer to obtain a final 2:1:1 diisocyanate:ionic monomer:polyol composition [22,23,24,25]. Also our group in previous works has investigated the structure–property relationship of 2:1:1 polyurethane ionomers using a variety of *soft* segments, including polypropylenoxide, polycaprolactone diol and poly-l-lactide diol [6,16,19,26], as a platform for the coordination of different metal ions. In the present work, within a framework of broadening this platform of polymers, several PU anionomers with different diisocyanates (aliphatic and aromatic) and two monomers’ molar ratios (2:1:1 and 3:2:1 diisocyanate:ionic monomer:polyol) were synthesized to investigate the effect of variable *hard* phase and ionic group content on PU *hard*/*soft* phase segregation and physical properties. PU anionomers were obtained starting from poly(tetrahydrofuran) as the *soft* phase, a carboxylated diol (di-hydroxymethyl propionic acid) as ionic monomer and different types of diisocyanates, either aliphatic or aromatic, as the *hard* phase. The obtained PUs were characterized in terms of water uptake, surface wettability and thermo-mechanical behavior. Furthermore, in light of the potential application of the synthesized materials in the biomedical field, polyurethanes were neutralized with metal ions, silver Ag(I) and zinc Zn(II), with the aim of providing polymers with intrinsic antimicrobial activity [27]. The obtained metal coordinated polymers were finally characterized in terms of their ability to inhibit the growth of two relevant clinical pathogens, *Staphylococcus epidermidis* and *Pseudomonas aeruginosa*.

## 2. Results and Discussion

Polyurethane ionomers are very versatile materials possessing polar groups, either cationic or anionic, in the polymer backbone. They have emerged as smart materials for a wide range of application fields, including biomedicine, especially in relation to the progress achieved in the production of new types of ionomers. The presence of ionic groups may affect polymer phase segregation as well as key material properties such as toughness, conductivity and hydrophilicity.

**Figure 1:**
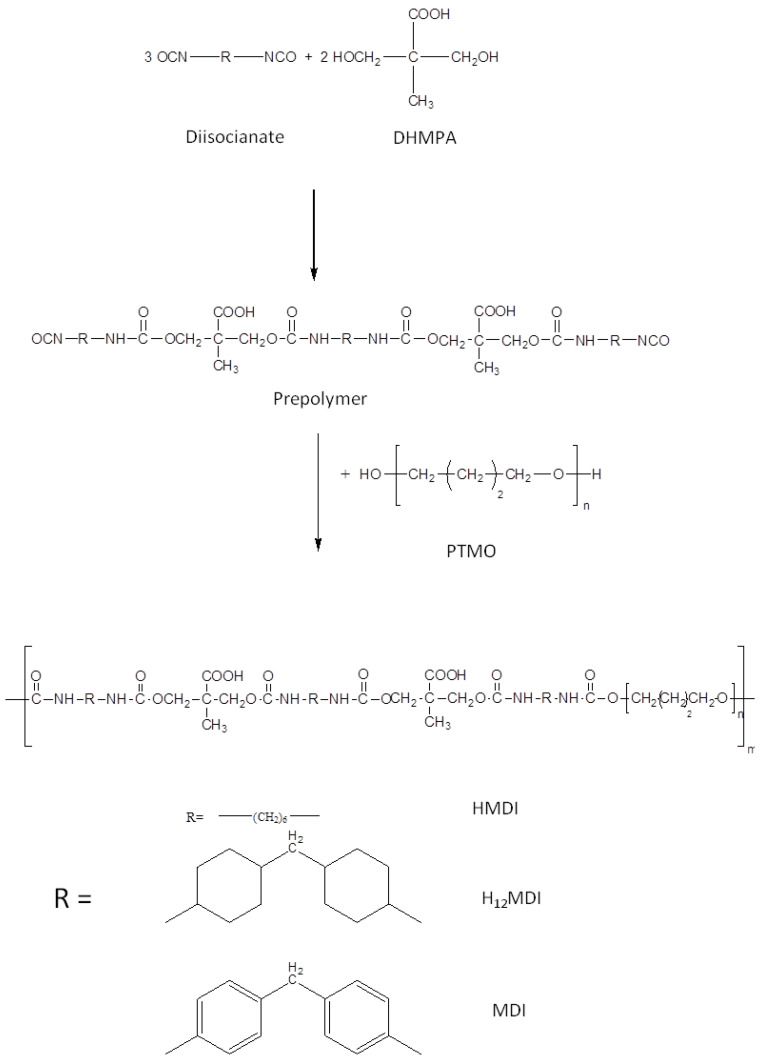
Stages of PU synthesis. Scheme for the diisocyanate:DHMPA:PTMO 3:2:1 molar ratio.

In this study, segmented PU anionomers were synthesized by a typical two-step polymerization reaction, keeping constant poly(tetrahydrofuran) (PTMO) as the *soft* phase while varying the diisocyanate as the main component of the *hard* phase. The use of a carboxylated diol, di-hydroxymethyl propionic acid (DHMPA), permitted to introduce carboxylic groups in the polymer *hard* phase, in a variable content depending on the monomers’ molar ratio employed during the synthesis. In Figure 1, a scheme of the polyurethanes’ synthesis is reported while in Table 1 the experimental conditions used during polymerization are reported together with the repeat unit composition, the polymer acronyms and the number of carboxylic groups per repeat unit.

Usually, segmented PUs are synthesized by firstly obtaining a prepolymer by reaction between the polyol and diisocyanate, and then the chain extender, generally bearing the ionic functionality, is added to increase the polymer molecular weight [22,23,24,25]. In contrast, in this study, in order to precisely control the composition of the *hard* segment, we decided to obtain first the *hard* segment (either DI-DHMPA-DI or DI-DHMPA-DI-DHMPA-DI), which was then reacted with the polyol PTMO. Indeed, since the effect of *hard* segment structure on PU physical properties is closely related also to preparation conditions, the obtainment of the *hard* segment with uniform chain length and controlled composition is needed to relate the behavior and properties of the final PUs to the different *hard* phases [28].

Polymerization success was confirmed by ^1^H-NMR spectroscopy (Supplementary Materials Figure S1) and gel permeation chromatography (GPC). In Table 2 the number average molecular weight (Mn), the weight average molecular weight (Mw) and the polydispersity (Ð) of polymers are reported.

Polymers possessed a number average molecular weight ranging from ca. 14 to 44 KDa (Table 2). PU1-MDI bearing the aromatic diisocyanate showed the highest molecular weight. In contrast, the lowest molecular weight was obtained by using the cyclic diisocyanate (PU2-H_12_MDI), suggesting an influence of reactivity of the employed diisocyanate on the extent of the polymerization reaction.

In general, the molecular weights of our polymers are higher than those reported in the literature for PUs having a similar 3:2:1 monomers’ ratio but obtained with a different polymerization procedure [21].

### 2.1. Study of Polymer Hard/Soft Phase Segregation

The *hard*/*soft* phase segregation of the synthesized PUs was investigated by different techniques, specifically FTIR spectroscopy, differential scanning calorimetry and dynamo-mechanical analysis. In general, the phase segregation in polyurethanes is influenced by several factors, including the polarity of monomers, length of *hard* and *soft* segments and *hard*/*soft* phase ratio.

**Figure 2:**
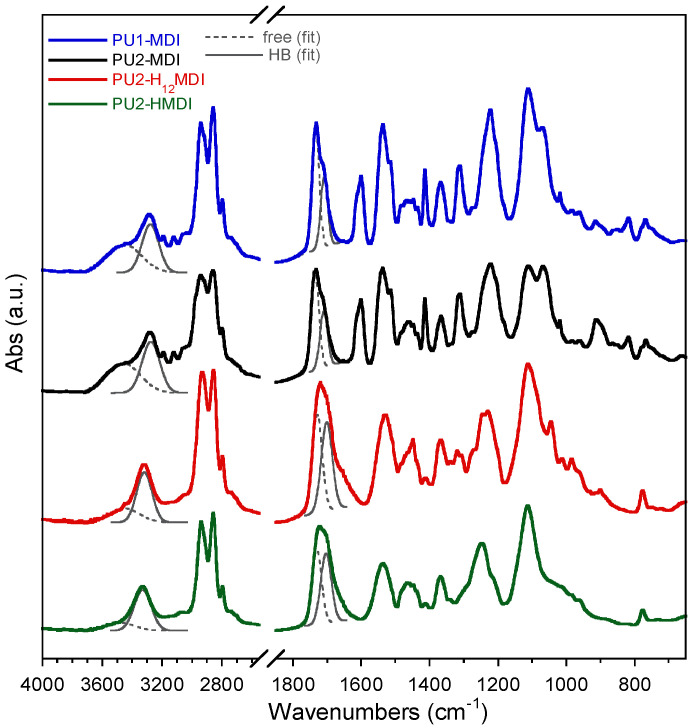
FTIR spectra of polyurethane ionomers. Band deconvolutions of the N-H stretching (3600–3200 cm^−1^) with two Gaussians centered at about 3460 cm^−1^ and 3300 cm^−1^ for free (ν N-H_free_) and hydrogen-bonded N-H (ν N-H_HB_) and of the C = O stretching (1800–1600 cm^−1^) with two Gaussians centered at about at 1730 cm^−1^ and at 1705 cm^−1^ for free (ν C = O_free_) and hydrogen-bonded C = O (ν C=O_HB_).

FTIR spectroscopy permits not only to evaluate the presence of specific functional groups but also to investigate if these groups are involved in hydrogen bonds [29]. Indeed, intermolecular interactions due to hydrogen bonding play a key role in the compatibility between the *hard* and *soft* segments, thus affecting the phase segregation and end-properties of polyurethanes. In polyurethanes, the main groups involved in hydrogen bonding are the urethane NH and C = O, the ether oxygens of the *soft* phase and, in our case, the carboxylic group. In Figure 2, the FTIR spectra of polymers are reported. In all of the spectra, the peak at 3300 cm^−1^ was related to NH stretching, the adsorption peaks in the 2800–3000 cm^−1^ range were attributed to the CH_2_ and CH_3_ stretching, the peak at ca. 1700 cm^−1^ to the stretching of the C = O group, the peak at 1540 cm^−1^ to NH bending and C-N stretching, the peaks at 1450 and 1370 cm^−1^ to CH_2_ and CH_3_ bending, and the peaks at 1100 cm^−1^ and at 1065 cm^−1^ to the stretching of the polyol C-O-C either free or involved in hydrogen bonding. In the spectra of PU1-MDI and PU2-MDI, possessing the aromatic diisocyanate MDI, a broad band in the 3400–3600 cm^−1^ range, presumably related to the stretching of NH groups not involved in hydrogen bonding, and a peak at 1600 cm^−1^ related to the stretching of the C = C of the aromatic ring were also present.

**Figure 3:**
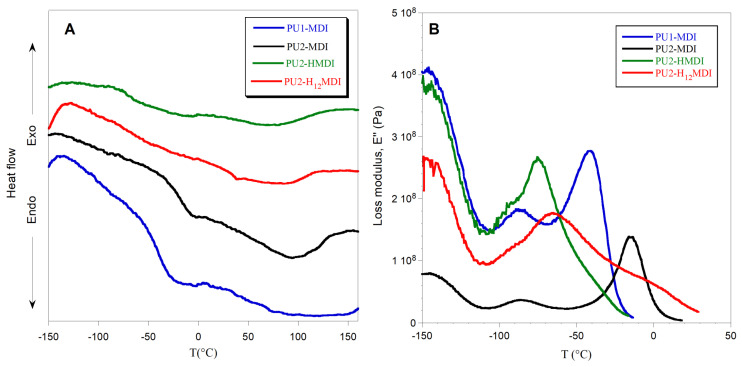
DSC thermograms (**A**) and loss modulus E″ (**B**) of polymers.

To have an estimation of *hard*/*soft* phase segregation, deconvolution of bands related to N-H stretching (3600–3200 cm^−1^) and C = O stretching (1800–1600 cm^−1^) was performed. The ratios between the area of the Gaussians (Figure 2) at 1705 cm^−1^ (C = O_HB_) and 1730 cm^−1^ (C=O_free_) and between the area of Gaussians at 3300 cm^−1^ (NH_HB_) and at 3460 cm^−1^ (N-H_free_) were determined and reported in Table 3. Since the urethane C=O of the *hard* phase can establish H-bonds also with NH and COOH groups always present in the *hard* phase, an increase in these two ratios suggests an increase in *hard*/*hard* phase interactions and thus an increase in *hard*/*soft* phase segregation [30]. As it can be observed in Table 3, the polymers PU1-MDI and PU2-MDI, containing the aromatic diisocyanate, showed area ratios lower, and then lower phase segregation, than PU2-H_12_MDI and PU2-HMDI, containing instead aliphatic diisocyanates. Such a finding was confirmed by differential scanning calorimetry (DSC) and dynamo-mechanical analysis (DMA). Specifically, the DSC thermograms and the loss modulus of polymers are reported in Figure 3A,B, respectively, while in Table 3, the glass transition temperature (T_g_), determined by both DSC and DMA, and the specific heat variation (ΔC_p_) are reported together with the T_g_ of the free *soft* phase, PTMO.

In the DSC thermograms (Figure 3A), a flex related to the glass transition temperature of the *soft* phase and a large endothermic band related to the melting of ordered portions of the polymer can be observed. In the DMA graph (Figure 3B), instead, two peaks of the loss modulus were observed: the α peak in correspondence with the polymer glass transition temperature and the β peak at ca. −90 °C, related to either a relaxation of the *hard* phase [31] or water molecules strongly bonded to the polymer chains [32].

In general, the glass transition temperature is a key parameter for studying polyurethane *hard*/*soft* phase segregation. Indeed, when the phase segregation is high, the *soft* phase has a good mobility, because it is not involved in H bonds with the *hard* phase, and shows a low value of T_g_, close to that of the free *soft* phase; in our case, PTMO. In agreement with FTIR findings, polymers with aliphatic diisocyanates, PU2-H_12_MDI e PU2-HMDI possessed very low T_g_ (Table 3), much lower than PU1-MDI and PU2-MDI, suggesting a strong *hard*/*soft* phase segregation. Between the two polymers possessing the aromatic diisocyanate (PU1-MDI and PU2-MDI), PU2-MDI had the highest T_g_ (Table 3), evidencing that the presence of a higher number of COOH groups in the *hard* phase promoted phase mixing.

The specific heat variation at the T_g_ (Table 3) further confirms the higher phase segregation of PUs having aliphatic diisocyanates. Indeed, PU2-H_12_MDI and PU2-HMDI showed a ΔC_p_ lower than PU1-MDI and PU2-MDI, evidencing a lower energy necessary for the *soft* phase at the glass transition, as a consequence of the *soft* phase’s greater mobility. The influence of aliphatic diisocyanates on phase segregation found in this study cannot be explained by thermodynamic factors. Indeed, since aliphatic diisocyanates should possess better chemical compatibility with the aliphatic polyether *soft* phase, they should have promoted polymer phase mixing. We hypothesized that kinetic factors could justify our evidences, the aliphatic *hard* phase possessing flexibility and mobility higher than the aromatic *hard* phase. This hypothesis is supported by the study of Li and coworkers [33], who similarly found a dependence of phase structure on kinetic factors in PUs with different diisocyanates.

### 2.2. Polymers’ Mechanical Properties

**Figure 4:**
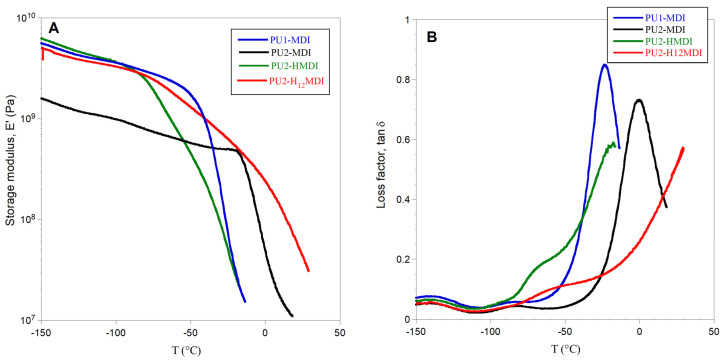
Storage modulus E′ (**A**) and loss factor tan δ (**B**) of polymers.

The mechanical properties of the polymers were investigated by DMA analysis. In Figure 3B, the loss modulus (E″) as a function of temperature is shown, while in Figure 4A,B the storage modulus E′ and the loss factor (tan δ), i.e., the ratio between the loss and storage moduli E″/E′, are reported, respectively.

All of the polymers showed a storage modulus E′ ranging from 2 × 10^9^ to 6 × 10^9^ Pa and, as expected, the less-segregated polymer PU2-MDI showed the lowest value. The polymer PU1-MDI showed an E′ value similar to PU2-HMDI and PU2-H_12_MDI, although less segregated, presumably because of the presence of the aromatic diisocyanate MDI which provides rigidity to the polymer. The abrupt decrease in E′ observed for all polymers with a temperature increase was associated with the polymer glass transition and occurred in correspondence with the α peak of the loss modulus E″ (Figure 4B).

As for loss factor tan δ, this parameter represents the energy dissipation potential of the material. Particularly, the height of tan δ is related to the mobile amorphous material, and then to the ability of the material to dissipate energy. As it can be observed in Figure 4B, a decrease in the viscous component involved in the glass transition was evident for PU2-MDI with respect to PU1-MDI. Presumably, the presence in PU2-MDI of a higher number of carboxylic groups which can establish H-bonds with the polyether PTMO imposes restrictions against the molecular motion of the *soft* phase itself. Unfortunately, for the aliphatic polymers PU2-HMDI and PU2-H_12_MDI, it was not possible to measure tan δ due to large sample creeping after the E″ peak.

### 2.3. Polymers’ Thermal Stability

**Figure 5:**
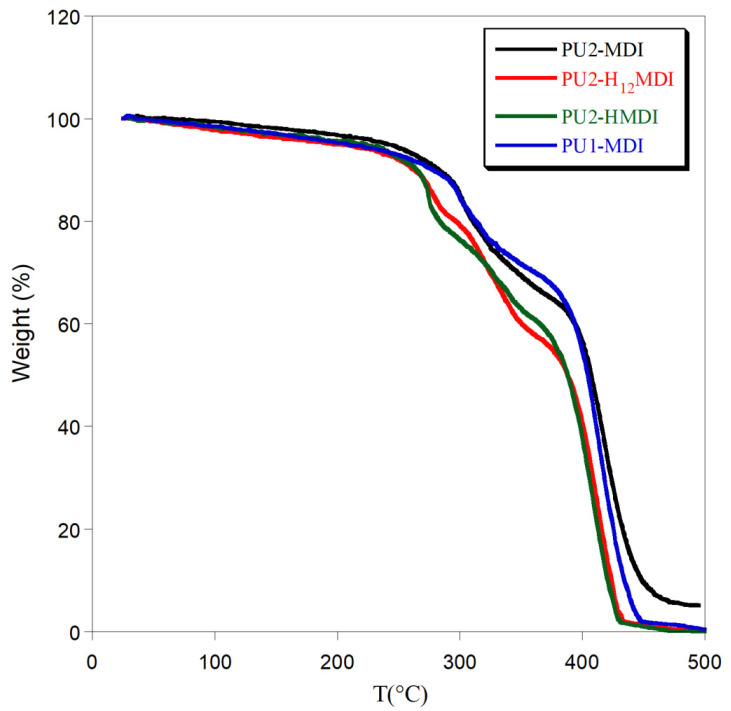
Thermogravimetric curves of polymers.

In Figure 5, the thermogravimetric curves of polymers are reported. All polymers showed a 5% initial weight loss, up to 150 °C, due to adsorbed water. Then, polymer degradation occurred between 250 and 450 °C by a multi-step process.

In general, the use of an aromatic diisocyanate increased polymer thermal stability. Indeed, PU1-MDI and PU2-MDI showed the first weight loss at ca. 310 °C while PU2-H_12_MDI and PU2-HMDI at ca. 280 °C. In addition, a decrease in polymer thermal stability with the increase in the *hard*/*soft* phase segregation was observed (PU2-MDI > PU1-MDI > PU2-H_12_MDI > PU2-HMDI). Presumably, an effect of mutual stabilization of the *soft* and *hard* segments in the less-segregated polymers can justify the observed thermal stability dependence [34].

### 2.4. Study of Polymer Hydrophilicity

**Figure 6:**
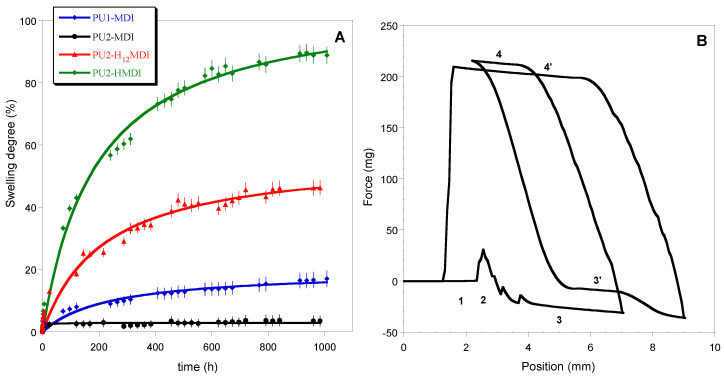
Curves of swelling in water (**A**); dynamic contact angle cycles of immersion for PU1-MDI when using the Wilhelmy plate method (**B**). In figure **B**, numbers 1 to 4 indicate the position of the plate with respect to the liquid: 1—Out of the liquid; 2—Point of touch of the sample with the liquid; 3—Immersion into the liquid (θ_adv_ I cycle); 4—Emersion from the liquid (θ_rec_ I cycle); 3’—Immersion into the liquid (θ_adv_ II cycle); 4’—Emersion from the liquid (θ_rec_ II cycle).

In Figure 6A, the kinetics of polymer swelling in water is reported. As for the thermal stability, also in this case, a correlation between the polymer phase segregation and swelling in water was found. Specifically, the most segregated polymers PU2-H_12_MDI and PU2-HMDI resulted in the most hydrophilic ones, reaching a swelling at the equilibrium of ca. 40% and 80%, respectively, after 30 days of immersion in water. Presumably, polymer phase segregation promoted the penetration of water molecules in the *soft* domains. In confirmation of that, PU2-MDI, although possessing two carboxylic groups per repeat unit, showed a swelling degree lower than PU1-MDI in relation to its lower phase segregation.

**Figure 7:**
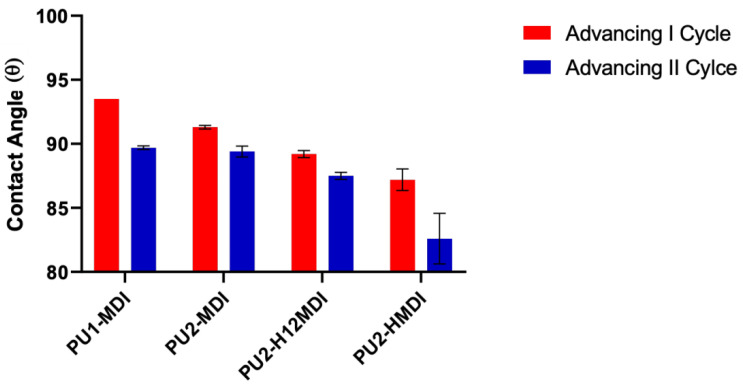
Advancing contact angles of polymers in the first and second cycle of immersion.

The measurements of dynamic contact angle (DCA) confirmed the higher hydrophilicity of polymers obtained with the aliphatic diisocyanates H_12_MDI and HMDI. In Figure 6B, as an example, DCA cycles of immersion for PU1-MDI when using the Wilhelmy plate method are reported, while in Figure 7, the advancing contact angles in the first and second cycle of immersion are reported for all of the polymers. As it can be observed, PU2-H_12_MDI and PU2-HMDI possessed a θ_adv_ lower than 90°, suggesting a certain polymer’s hydrophilicity, while PU1-MDI and PU2-MDI were hydrophobic polymers (θ_adv_ > 90°). In addition, all of the polymers showed a kinetic contact angle hysteresis evidenced by the decrease in the contact angle in the second cycle of immersion (Figure 7). Such a finding suggests a molecular rearrangement of the polymer surface on wetting, presumably involving the exposition of the hydrophilic *soft* phase at the polymer/water interface. Phase segregation may favor the mobility of the *soft* segments. The migration of polar groups towards hydrophilic environments was first reported by Blodgett and Langmuir [35], by studying the behavior of surfactant molecules. Such re-organization was later shown to be driven by the minimization of the surface free energy at the interface [36].

The increased polymer hydrophilicity observed especially in the most-segregated polyurethanes PU2-H_12_MDI and PU2-HMDI is definitely in favor of an application of the polymers in the biomedical field since it may promote polymer hemocompatibility [37] and provide the polymer with intrinsic protection to biofouling [26,38] throughout a surface-hydration mechanism [39].

### 2.5. Neutralization of Polymers with Metal Ions

The obtained polymers were finally neutralized with either silver or zinc ions in order to provide them with antimicrobial activity. There is by now a consolidated knowledge about the antimicrobial activity of several metals, especially silver, whose medicinal use has been prevalent until the discovery of antibiotics. Nowadays, the use of antimicrobial metals is experiencing a real renaissance also in relation to the worldwide-growing multidrug-resistance issue. Among metals, silver has been the most investigated metal due to its broad spectrum of activity coupled with a low propensity to induce antimicrobial resistance in bacteria [40]. Metal ions affect microbial viability by multiple mechanisms [41], including oxidative stress [42], protein dysfunction [43] or membrane damage [44].

Conventional approaches to obtain antimicrobial polymers based on silver or other metals are based on either the deposition of a metallic layer on a polymer surface [45] or entrapping metal nanoparticles [46]. In contrast, in this study, the coordination of polymers was obtained by simply dipping the synthesized PU ionomers sodium salts in an aqueous solution containing either silver or zinc salts. In this way, the active species of metals, Ag+ and Zn2+, were stably linked to the polymer backbone by exchanging with Na+ cation. ICP-AES analysis confirmed the ability of polymers to coordinate silver and zinc. Indeed, a complete saturation of carboxylic groups was observed when polymers were treated with silver salt while only 75% of carboxylic groups were neutralized with the bivalent Zn ion.

**Figure 8:**
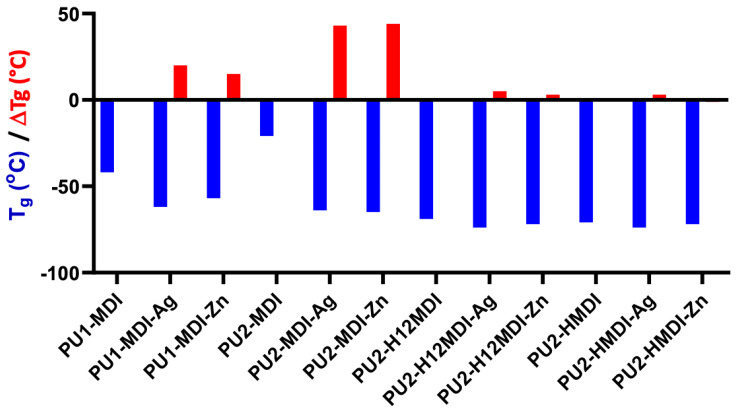
Glass transition temperature (T_g_) of polymers and decrease in T_g_ (red bars), ΔT_g_ = T_gPU_—T_gPU/metal_, after polymer neutralization with either silver or zinc.

The formation of PU/metal hybrids affected significantly the polymer *hard*/*soft* phase segregation. In fact, a decrease in polymer glass transition temperature was observed (Figure 8, red bars), especially for the less-segregated polymers PU1-MDI and PU2-MDI. 

Presumably, the neutralized carboxylic groups present in the *hard* phase tend to aggregate, thus favoring the cohesion of the *hard* domains, enhancing phase segregation and promoting the mobility of the *soft* segments (decrease in polymer T_g_).

Eisenberg and colleagues [47] first reported that, in ionomers, ionic groups tend to aggregate, leading to the formation of small aggregates containing a few ion pairs, called multiplets. Such multiplets act as ionic cross-links, reducing the mobility of the polymer chain fraction interested by the multiplets themselves. Han and Williams [48] proposed, for poly(ethylene–methacrylic acid) ionomers neutralized with different types of metal ions, the existence of two potential aggregation mechanisms and models: a cluster–multiplet model and a coordinated complex model.

**Figure 9:**
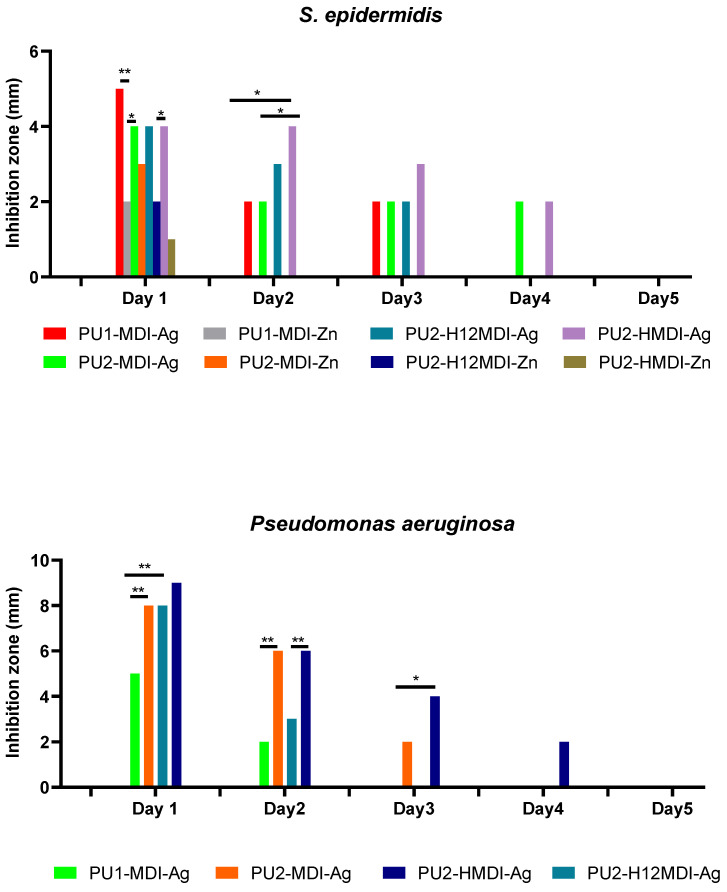
Inhibition halo measured around polymer disks against *S. epidermidis* and *P. aeruginosa* over time. The durability of polymer antimicrobial activity was evaluated by re-plating polymer disks every 24 h over a 5-day period. Statistical analysis showed a significant difference when * *p*-value < 0.05 and ** *p*-value < 0.01.

The antimicrobial activity of polymer/metal systems was tested against two bacterial strains, *S. epidermidis* and *P. aeruginosa*, chosen among the most relevant clinical pathogens. Specifically, the inhibition halo observed, after overnight incubation, around polymer disks placed onto agar plates previously seeded with the microorganisms was determined. As it can be observed in Figure 9, polymer/Ag systems showed a good antimicrobial activity towards both *S. epidermidis* and *P. aeruginosa* while polymer/Zn systems did not show any activity towards *P*. *aeruginosa*. As for *S. epidermidis*, silver was an antimicrobial agent better than Zn. In particular, on day 1, all of the polymer/Ag matrices showed inhibition halos higher than the polymer/Zn counterparts (Figure 9).

In addition, all of the polymer/Zn systems lost activity at day 2 while polymer/Ag hybrids showed a prolonged activity, which for PU2-MDI-Ag and PU2-HMDI-Ag lasted for 4 days. Interestingly, when tested towards the Gram-negative *P. aeruginosa*, silver/PU polymers showed inhibition halos higher than those registered towards *S. epidermidis*. This result is consistent with recent findings reporting the activity of colloidal silver against 270 strains, including Gram-negative and Gram-positive bacteria [49]. The authors showed how silver was able to significantly increase the production of reactive oxygen species (ROS) in Gram-negatives with respect to Gram-positives at 24 h incubation.

Finally, as expected, with the same type of diisocyanate, polymers with a higher content of ionic groups showed a longer antimicrobial activity. For instance, the activity of PU2-MDI-Ag against *S. epidermidis* lasted 3 days versus 2 days of PU1-MDI (Figure 9). This finding can be definitely related to the more or less marked ability of polymers to coordinate metal ions in dependence on the ionic groups’ concentration.

## 3. Materials and Methods

### 3.1. Materials

4,4′-methylenebis(phenyl isocyanate) (C_15_H_10_N_2_O_2_, MDI, Sigma Aldrich, St. Louis, MO, USA) was distilled before use. Poly(tetrahydrofuran) (PTMO) (1400 g mol^−1^, Sigma Aldrich) was degassed under vacuum at 60 °C for 12 h. 2,2-bis(hydroxymethyl) propionic acid (DHMPA, Sigma Aldrich), hexamethylene diisocyanate (C_8_H_12_N_2_O_2_, HMDI, Sigma-Aldrich) and 4,4′-Methylenebis(cyclohexyl isocyanate) (C_15_H_22_N_2_O_2_, H_12_MDI, Sigma-Aldrich), were used as received. The solvents dimethyl formamide (DMF, Sigma-Aldrich) and 2-pentanone (PENT, Sigma-Aldrich) were used as received.

### 3.2. Synthesis of Ionomer Polyurethanes

Carboxylated segmented polyurethanes (PUs) were synthesized by a step-growth polyaddition of a diisocyanate (MDI, HMDI or H_12_MDI), a carboxylated diol (DHMPA) and a macrodiol (PTMO) (Figure 1). In order to obtain PUs with either one or two carboxyl groups per repeat unit, two diisocyanate:DHMPA:PTMO stoichiometric ratios were used, 2:1:1 and 3:2:1, respectively.

In Table 1, the experimental conditions of the two stages of the polymerization reaction are reported. Briefly, in the first stage, the diisocyanate was dissolved in anhydrous DMF, under nitrogen flow. Then, the tributylamine (TBA) salt of DHMPA was dissolved in a 2/1 DMF/PENT mixture and added to the reaction in either a 2:1 or 3:2 Diisocyanate:DHMPA stoichiometric ratio. The reaction was carried out at temperatures for a time suitable to have either half (Diisocyanate:DHMPA 2:1) or 2/3 (Diisocyanate:DHMPA 3:2) of the initial NCO groups still unreacted (Table 1). The unreacted NCO groups at determined times were evaluated by titration with hydrochloric acid (0.1 M) after reaction with an excess of di-n-butylamine.

The obtained prepolymers were then reacted with PTMO, previously degassed and dissolved in anhydrous DMF. In this second stage, the dibutyl tin dilaurate was added as the catalyst. After 24 hr of polymerization, the polymer was recovered by precipitation in NaOH 0.1 N to obtain the polymer sodium salt. The polymer was then purified by Soxhlet extraction in diethylether to eliminate TBA and treated with HCl to restore the polymer acidic groups. Finally, polymers were dried in a vacuum oven at 50 °C and stored. The obtained polyurethanes were named PUX-D, where X is the number of COOH per repeat unit and D stands for the diisocyanate used in the synthesis, either MDI, HMDI or H_12_-MDI (Table 1).

### 3.3. Preparation of Metal-Decorated Polyurethanes 

To prepare metal-decorated polyurethanes, the sodium salts of polyurethanes were dissolved in water and treated with equimolar amounts of either AgNO_3_ or Zn(NO_3_)_2_ solutions. The resulting precipitate was recovered, dissolved in N,N-DMF and poured on Teflon plates to obtain films after solvent evaporation. In order to avoid the oxidation of PU/metal systems, polymers were kept in the dark. 

Inductively Coupled Plasma Atomic Emission Spectrometry (ICP-AES, VARIAN) was employed to determine the number of ions present in the polymers by checking peak intensities at the following wavelengths: λ_Ag_ = 328 nm and λ_Zn_ = 206 nm.

### 3.4. Polymer Characterization

#### 3.4.1. Spectroscopic Analysis

^1^H-NMR spectra were performed employing a Varian XL 300 instrument and deuterated di-methylformamide (DMF-d7) as the solvent.

Fourier Transform Infrared spectroscopy (FTIR) analysis was performed in an Attenuated Total Reflection (ATR) by a Nicolet 6700 (Thermo Fisher Scientific, Waltham, MA, USA) equipped with a Golden Gate ATR accessory, at a resolution of 2 cm^−1^ and co-adding 100 scans. Band deconvolution was performed with the Peak resolve function included in Omnic 8.3 software by Thermo Fisher Scientific. The N-H stretching region (3600–3200 cm^−1^) was fitted by using two Gaussian functions, centered at about 3460 cm^−1^ and 3300 cm^−1^ for free (ν N-H_free_) and hydrogen-bonded N-H (ν N-H_HB_), respectively. Moreover, the C = O stretching region (1800–1600 cm^−1^) was fitted with three Gaussian profiles, assigned to the free (ν C = O_free_) at 1730 cm^−1^ and the hydrogen-bonded moiety (νC = O_HB_) at 1705 cm^−1^, as well as an additional band at 1660 cm^−1^.

#### 3.4.2. Gel Permeation Chromatography

Gel permeation chromatography (GPC) was carried out at 30 °C in HPLC grade chloroform at a 1 mL/min by using a 150-C Waters GPC apparatus equipped with a differential refractive index detector. Monodisperse polystyrene samples with molecular weights ranging from 1.3 × 10^3^ to 1.5 × 10^6^ g/mol were used as standards. Two cross-linked polystyrene (PS) columns (Water Ultrastyragel) with a separation range of 2 × 10^3^–1 × 10^6^ g/mol (linear) and 200–3 × 10^4^ g/mol were used.

#### 3.4.3. Thermal Analysis

Differential scanning calorimetry (DSC) was performed by a Mettler TA-3000 DSC apparatus. Thermograms were acquired at 10 °C min^−1^ in the −150 to +150 °C temperature range, under N_2_ flux. Thermogravimetric analysis (TGA) was carried out by employing a Mettler TG 50 thermobalance at a heating rate of 10 °C min^−1^ under N_2_ flow from 25 to 500 °C.

#### 3.4.4. Polymer Swelling Ability

Swelling experiments were performed in water at room temperature on round-shaped polymer films (1 cm in diameter and 100 µm in thickness), obtained by casting a 5% polymer solution in tetrahydrofuran (THF) on Teflon plates, followed by solvent evaporation at room temperature.

The polyurethane disks were immersed in water and, at increasing times, disks were removed from water and weighed, after removal of the excess of solvent using filter paper. The swelling degree, SD, was calculated by applying the following equation:

(1) $$SD\;\left(\%\right)=\;\frac{W_{t}-\;W_{0}}{W_{0}}\;\times \;100$$

where *W_t_* is the weight of the sample after swelling at time *t* and *W*_0_ is the initial weight of the film. Five parallel swelling experiments were performed for each sample and data were reported as average value ± standard deviation.

#### 3.4.5. Dynamic Contact Angle

Polymer surface wettability was determined by measuring the dynamic contact angle (DCA) using the Wilhelmy method. DCA measurements were performed by a Dynamic Contact Angle Analyzer Cahn (CAHN DCA 312), on glass slides (1 cm × 2 cm) previously coated with a thin polymer layer by solvent casting. Two consecutive immersion cycles were carried out at a 70 µm/s immersion rate. In the second immersion cycle, the sample immersed area was greater than that of the first immersion cycle in order to verify the absence of any leaching from sample to water.

The advancing contact angle (θ_adv_) was recorded during the immersion phase both in the first and second immersion cycle, while the receding contact angle (θ_rec_) was measured during the emerging phase.

#### 3.4.6. Mechanical Analysis

Dynamo-mechanical analysis (DMA) of polymers was performed by a Rheometrics Scientific Analyzer RSA II. Polymer specimens were rectangular films with sizes of 30 × 1 × 0.1 mm, length by width by thickness. The real (*E*′) and imaginary (*E*″) components of complex modulus E* were investigated by heating the sample at 5 K/min in the range from −150 to 50 °C, at a test frequency of 1 Hz and a strain limit of 0.2%.

### 3.5. Assessment of Antimicrobial Activity of Polymer/Metal Systems

The antimicrobial activity of polymer/metal systems was tested by a modified Kirby–Bauer test against the Gram-negative *Pseudomonas aeruginosa* (ATCC 27853) and the Gram-positive *Staphylococcus epidermidis* ATCC 35984, oxacillin-resistant and exopolysaccharide strong producer [50].

For the Kirby–Bauer test, polymer disks (1 cm in diameter) were placed on TSA plates previously seeded with 10^8^ CFU/ml (0.5 McFarland) of either *S. epidermidis* or *P. aeruginosa*. Following incubation at 37 °C for 24 h, the diameters of inhibition halos of bacterial growth around the discs were measured. To assess the durability of the antimicrobial activity, disks were re-tested every 24 h under the same conditions over a period of 5 days.

### 3.6. Statistics

Analysis of variance comparisons were performed using MiniTab. Differences were considered significant for *p* values of <0.05. Data were reported as means ± 1 SD. 

## 4. Conclusions

Polyurethane ionomers are considered promising materials for a wide range of applications due to their segregated structure which confers tunable physico-chemical properties. In this study, polyurethane ionomers with different *hard* segments and concentration of ionic groups per polymer repeat unit were synthesized. Findings showed that, when aromatic diisocyanate was employed, the increase in ionic group content promoted polymer *hard*/*soft* phase mixing, presumably because of the establishment of hydrogen bond interactions between the carboxylic groups of the *hard* segment and the polyether *soft* phase. With the same content of carboxylic groups, instead, the presence of aliphatic diisocyanates in the polymers promoted *hard*/*soft* phase segregation. Kinetics factors may be responsible for such a finding, the aliphatic diisocyanates possessing flexibility and mobility higher than aromatic ones. An increase in *hard*/*soft* phase segregation resulted in a significant improvement in polymer bulk and surface hydrophilicity, key features for the successful application of polymers in biological environments. The neutralization of polymer ionic groups with metal ions, silver and zinc, permitted the obtaining of metal coordination polymers with interesting antimicrobial features towards Gram-positive and Gram-negative bacteria. Additionally, polymer phase segregation was affected by metal coordination, which presumably induced the formation of ionic multiplets increasing *hard* segments’ cohesion.

Although the herein-reported investigation concerns basic research mainly designed to increase scientific knowledge on the structure–property relationship in polyurethane ionomers, a potential application of the developed polymers as antimicrobial coatings for different kinds of surfaces, including medical devices and fabric wound dressings, can be envisaged on the basis of their interesting antimicrobial features. In this regard, additional biological tests are planned to be performed in the near future to assess the antimicrobial efficacy of polymers also in in vitro models resembling in vivo experimental conditions.

## Figures and Tables

**Table 1:** Experimental conditions of polyurethanes’ synthesis, acronym and repeat unit composition of the synthesized polymers.

PUX-D Polymer	Repeat Unit Composition	Experimental Conditions	COOH Per Repeat Unit
**PU1-MDI**	2 MDI: 1 DHMPA: 1 PTMO	I stage: T = 25 °C, t = 30 min II stage: T = 25 °C, t = 24 h	1
**PU2-MDI**	3 MDI: 2 DHMPA: 1 PTMO	I stage: T = 25 °C, t = 30 min II stage: T = 25 °C, t = 24 h	2
**PU2-H_12_MDI**	3 H_12_MDI: 2 DHMPA: 1 PTMO	I stage: T = 50 °C, t = 60 min II stage: T = 70 °C, t = 24 h	2
**PU2-HMDI**	3 HMDI: 2 DHMPA: 1 PTMO	I stage: T = 50 °C, t = 50 min II stage: T = 70 °C, t = 24 h	2

**Table 2:** Molecular weight and polydispersity index (Ð) of polymers.

PUX-D Polymer	Mn (g/mol)	Mw (g/mol)	Ð
**PU1-MDI**	44,500	88,300	1.98
**PU2-MDI**	32,700	50,000	1.53
**PU2-H_12_MDI**	14,500	24,300	1.68
**PU2-HMDI**	39,600	71,500	1.81

**Table 3:** FTIR ratio between peaks at 1710 cm^−1^ (C=O_HB_) and 1730 cm^−1^ (C = Of_ree_) and peaks at 3300 cm^−1^ (NH_HB_) and at 3460 cm^−1^ (N-H_free_), glass transition temperature (T_g_), determined by differential scanning calorimetry (DSC) or dynamo-mechanical (DMA) analysis, and specific heat variation (ΔC_p_). PTMO is the *soft* phase.

PUX-D Polymer	FTIR A_C = O HB_/A_C = O free_	FTIR A_N-H HB_/A_N-H free_	T_g_ (°C) DSC	T_g_ (°C) DMA	ΔC_P_ (J/g °C) DSC
**PU1-MDI**	0.56	0.86	−42 °C	−41 °C	0.39
**PU2-MDI**	0.56	0.92	−21 °C	−15 °C	0.36
**PU2-H_12_MDI**	1.00	1.87	−69 °C	−66 °C	0.31
**PU2-HMDI**	1.06	3.11	−71 °C	−75 °C	0.29
**PTMO**	−	−	−84 °C	−	−

## Data Availability

Data is contained within the article or Supplementary Materials.

## Supplementary Materials

The following are available online at https://www.mdpi.com/article/10.3390/ijms22116134/s1.
