# Supplementary material for: Silver- and Zinc-Decorated Polyurethane Ionomers with Tunable Hard/Soft Phase Segregation
Source: Int J Mol Sci. 2021 Jun 7;22(11):6134. doi: 10.3390/ijms22116134 (PMC8200980; doi:10.3390/ijms22116134)
Supplement: Supplementary file 1 [file ijms-22-06134-s001.zip › ijms-1233037-SI.pdf]

Silver and zinc decorated-polyurethane ionomers with tunable *hard/soft* phase segregation

Irene Rapone[1], Vincenzo Taresco[2], Valerio Di Lisio[1], Antonella Piozzi[1], Iolanda Francolini[1]*

[1] Department of Chemistry, Sapienza University of Rome, Rome, Italy

[2] School of Chemistry, University of Nottingham, Nottingham, UK

*** Correspondence to: Prof. Iolanda Francolini**

Department of Chemistry, Sapienza University of Rome

Piazzale Aldo Moro, 5 – 00185, Rome, Italy

E-mail: **iolanda.francolini@uniroma1.it**

Phone: **+39 06 49913162**

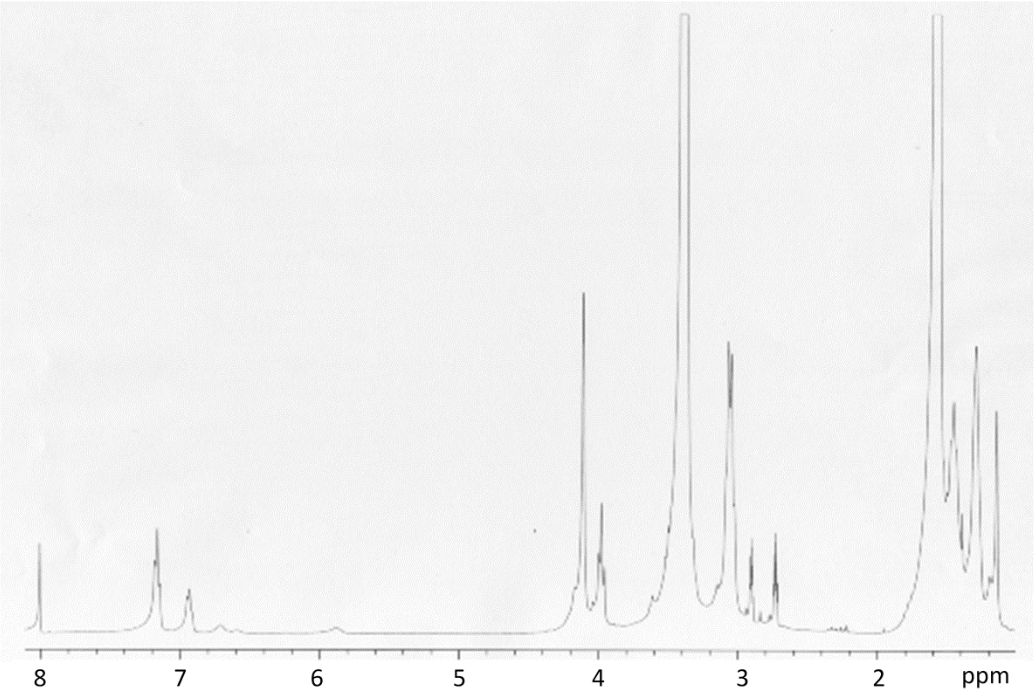

Figure S1: ^{1}H-NMR spectrum of PU2-HMDI in N,N-DMFd$_7$.

The ^{1}H-NMR spectrum of PU2-HMDI shows the characteristic peaks that in accordance with the proposed structure confirmed the success of the reaction. In particular, Signals at 6.9 and 7.2 ppm were related to urethane NH, specifically to the *syn* and *anti* rotamers around the C-N linkage of the urethane group [29, 30]. Signals at 1.6 and 3.5 ppm were related to the hydrogens of the internal CH_2 of HMDI and PTMO while the signal at 3.1 ppm was attributed to CH_2 of HMDI when linked to the urethane NH. Finally, signals at 4.1 and 1.1 ppm were related to the CH_2 and CH_3 of DHMPA, respectively. The 3:2:1 monomer molar ratio of the polymer repeat unit was confirmed by the integrals' ratio of signals at 6.9 (urethane NH) and 1.1 (DHMPA CH_3) ppm.
